# Clinical Characteristics, Surgical Risks, and Long-Term Prognosis of Good's Syndrome

**DOI:** 10.7759/cureus.111036

**Published:** 2026-06-17

**Authors:** Shinya Otsuka, Haruhiko Shiiya, Akihiro Sasaki, Kazuto Ohtaka, Aki Fujiwara-Kuroda, Masato Aragaki, Kanako C Hatanaka, Tatsuya Kato

**Affiliations:** 1 Department of Thoracic Surgery, Hokkaido University Hospital, Sapporo, JPN; 2 Center for Development of Advanced Diagnostics, Hokkaido University Hospital, Sapporo, JPN

**Keywords:** good's syndrome, infectious disease control, intravenous immunoglobulins (ivig), pure red cell aplasia (prca), robotic thymectomy

## Abstract

Objective

Good's syndrome (GS), a rare condition characterized by thymoma with hypogammaglobulinemia, is associated with recurrent infections and poorer prognosis. Advances in minimally invasive procedures, such as video-assisted thoracoscopic surgery (VATS) and robotic-assisted thoracoscopic surgery (RATS), may help reduce perioperative complications in thymoma surgery. Given these considerations, we aimed to evaluate the surgical outcomes and long‑term prognosis of patients with GS undergoing thymectomy, with particular attention to minimally invasive approaches, such as VATS and RATS.

Methods

We retrospectively reviewed thymectomy cases at Hokkaido University Hospital (2007-2024). Clinical background, surgical procedures, and outcomes were compared between GS and non-GS patients. Survival was analyzed using Kaplan-Meier curves and log-rank tests. We also conducted a detailed evaluation of perioperative management and long-term outcomes in the seven patients with GS.

Results

Among 104 thymectomy cases, seven had GS. All GS patients had type AB thymoma, significantly more frequent than in non-GS cases. Surgical approaches included median sternotomy (n=2), VATS (n=2), and RATS (n=3). Perioperative outcomes were comparable to those of non-GS patients. However, immunodeficiency persisted, and five GS patients developed severe late postoperative infections, leading to two deaths. Additional late complications included extrathymic malignancies (n=2) and pure red cell aplasia (n=3). Despite the limited sample size, overall survival was significantly worse in GS patients (p=0.002).

Conclusions

Thymectomy for GS can be performed safely, including VATS and RATS. Nevertheless, long-term prognosis remains poor because of persistent immunodeficiency, leading to severe infections and secondary malignancies, highlighting the need for vigilant postoperative management.

## Introduction

Good's syndrome (GS) is a rare disorder characterized by thymoma associated with hypogammaglobulinemia. Due to severe infections associated with immunodeficiency, patients with GS generally have a poorer prognosis compared to those with typical thymoma. Complete tumor resection and appropriate infection management are crucial for long-term survival [1-4].

Recently, surgical intervention for thymoma has advanced significantly. Video-assisted thoracoscopic surgery (VATS) and robotic-assisted thoracoscopic surgery (RATS) have been reported to have a lower risk of perioperative infections compared to traditional thoracotomy, with equivalent rates of complete resection [5-7]. These developments suggest that minimally invasive thymectomy may also be feasible for patients with GS, although evidence remains limited. To date, only a small number of reports have described the surgical management or long-term clinical course of GS, and data comparing GS with non-GS thymoma patients are scarce. A clearer understanding of perioperative safety, postoperative complications, and long-term outcomes in GS could help optimize treatment strategies and follow-up care. Evidence from Japanese patients with GS is scarce, and additional clinical data may help improve understanding of this condition.

Since 2007, we have performed thymectomy in seven patients with GS. By analyzing these cases in comparison with thymoma patients without GS, we conducted a study to clarify the clinical characteristics and surgical outcomes associated with this condition. The primary objective of this study was to evaluate the perioperative safety and long‑term prognosis of thymectomy in patients with GS. As a secondary, exploratory objective, we aimed to assess the feasibility and outcomes of minimally invasive approaches, including VATS and RATS, compared with conventional open surgery.

This article was previously presented as a meeting abstract at the 2025 World Conference on Lung Cancer on September 6, 2025.

## Materials and methods

Ethical considerations

This study was conducted in compliance with the principles of the Declaration of Helsinki and was approved by the Ethics Committee of Hokkaido University Hospital (approval number: 024-0445) on February 26, 2025. Written informed consent to publish was obtained from all of the participants.

Patients

Data were retrospectively collected from patients who underwent curative surgery for thymoma at Hokkaido University Hospital between July 2007 and January 2024. Patients with neuroendocrine tumors, thymic carcinoids, or distant metastases were excluded from the study.

Background data (age, sex, comorbid autoimmune diseases), clinical data (pathologic tumor size, histological type, TNM (tumor, nodes, and metastasis) classification [8], Masaoka-Koga stage [9]), and perioperative data (surgical procedure, surgical approach, blood loss, operation time, perioperative complications) were collected from medical records. Post-discharge data were collected by reviewing patient records at our hospital or contacting the physicians at other institutions where follow-up was conducted.

For cases with a history of recurrent infections, preoperative tests for IgG, IgA, and IgM were performed. Patients who exhibited significant reductions across all immunoglobulin classes were diagnosed with GS. No other causes of secondary hypogammaglobulinemia were identified in these patients. In selected cases, urinary Bence-Jones protein and serum IgD and IgE levels were measured to exclude lymphoid malignancies, such as multiple myeloma. Cellular immunodeficiency was also ruled out by evaluating the absolute CD4⁺ T‑cell count. All patients diagnosed with GS at our institution during the study period underwent surgery. The surgical procedure (partial, total, or extended thymectomy) was determined based on the tumor size on radiographic examination, the presence of invasion into adjacent organs, and the coexistence of myasthenia gravis (MG). Until September 2019, the surgical approach (median sternotomy or VATS) was selected based on the aforementioned evaluations, the surgeon's experiences, and level of expertise. After that period, RATS was gradually introduced for cases with smaller tumor sizes, and it became the primary choice in principle. Overall survival (OS) was defined as the time from surgery to last follow-up or death from any cause. Surgical complications were classified according to the Clavien-Dindo classification [10]. The pathological stage was analyzed based on Masaoka-Koga classification and the ninth edition of the TNM classification.

Perioperative and long‑term management

According to our institutional protocol, cefazolin (1 g) was administered within the standard pre‑incision prophylactic window and repeated 3 hours later. Antibiotics, including intravenous cefazolin or oral agents, were continued for up to two weeks postoperatively, depending on clinical signs such as fever or suspected surgical site infection.

Serum immunoglobulin (IgG, IgA, and IgM) levels were measured within one month prior to surgery, and intravenous immunoglobulin (IVIG) was administered within one week before the operation when deemed necessary. The IVIG dose was adjusted to maintain serum IgG levels at or above 600-700 mg/dL and was determined within the insurance‑approved range for the treatment of hypogammaglobulinemia.

Postoperatively, patients were followed in the outpatient clinic approximately every one to two months. Serum immunoglobulin levels were monitored at each visit, and IVIG dosing was modified accordingly. Detailed interviews were conducted to assess physical symptoms, and if any signs suggestive of infection or autoimmune disease were identified, comprehensive laboratory and radiologic evaluations were performed, including targeted assessments for cytomegalovirus and invasive fungal infections. Whole‑body CT scans were obtained every 6-12 months to assess for thymoma recurrence and to screen for secondary malignancies.

Statistical analysis

Continuous variables were presented as median and interquartile range and compared using the Mann-Whitney U test between GS and non-GS patients. Categorical data were expressed as frequencies (percentages) and compared using Fisher's exact test. Blood loss of less than 5 mL was considered as 0 mL in the analysis.

Survival curves were estimated using the Kaplan-Meier method for patients with GS and non-GS, and compared using the log-rank test. We performed univariate logistic regression analyses to assess the impact of tumor diameter on various perioperative complications. For these analyses, data from all surgical approaches were integrated into a single cohort. The odds ratio and 95% confidence interval were calculated for each 1-mm increase in tumor diameter. Given the small number of GS cases, multivariable analysis was not performed. Statistical analyses were performed using R (version 4.3.3; R Development Core Team, Vienna, Austria), and statistical significance was defined as p < 0.05.

## Results

Patient characteristics

The clinical features of the patient cohort are summarized in Table 1. A total of 104 cases with thymoma were included in the study, consisting of 97 non-GS cases and 7 GS cases. There were no notable differences in age (p=0.94) or sex (p=0.08) between the two groups. Although tumor size tended to be larger in the GS group (p=0.02), there were no significant differences in TNM stage (p=0.64) or Masaoka-Koga classification (p=0.92). All GS cases were associated with type AB thymoma (p=0.02).

**Table 1:** Clinical features of the cases with thymoma Continuous variables are presented as medians and IQR. Counts are given as numbers and percentages. Abbreviations: GS, Good's syndrome; IQR, Interquartile range; MS, Median sternotomy; VATS, Video-assisted thoracoscopic surgery; RATS, Robotic-assisted thoracoscopic surgery

Characteristics		Overall (n=104)	Non-GS (n=97)	GS (n=7)	p-value
Age, median (IQR)		63.0 (53.0, 70.2)	63.0 (53.0, 71.0)	62.0 (57.0, 67.5)	0.94
Sex, n (%)	Female	56 (53.8)	55 (56.7)	1 (14.3)	0.08
	Male	48 (46.2)	42 (43.3)	6 (85.7)	
Approach, n (%)	MS	33 (31.7)	31 (32.0)	2 (28.6)	0.83
	VATS	37 (35.6)	35 (36.1)	2 (28.6)	
	RATS	34 (32.7)	31 (32.0)	3 (42.9)	
Tumor size (mm), median (IQR)		55.0 (30.0, 75.0)	51.0 (30.0, 75.0)	70.0 (63.5, 92.5)	0.02
Histological type, n (%)	A	12 (11.5)	12 (12.4)	0	0.02
	AB	35 (33.7)	28 (28.9)	7 (100)	
	B1	11 (10.6)	11 (11.3)	0	
	B2	22 (21.2)	22 (22.7)	0	
	B3	5 (4.8)	5 (5.2)	0	
	Thymic cancer	15 (14.4)	15 (15.5)	0	
	Others	4 (3.8)	4 (4.1)	0	
TNM classification, n (%)	I	85 (81.7)	78 (80.4)	7 (100)	0.64
	II	6 (5.8)	6 (6.2)	0	
	IIIa	3 (2.9)	3 (3.1)	0	
	IVa	10 (9.6)	10 (10.3)	0	
Masaoka-Koga classification, n (%)	I	30 (28.8)	27 (27.8)	3 (42.9)	0.92
	IIa	40 (38.5)	38 (39.2)	2 (28.6)	
	IIb	14 (13.5)	13 (13.4)	1 (14.3)	
	III	11 (10.6)	10 (10.3)	1 (14.3)	
	IVa	8 (7.7)	8 (8.2)	0	
	IVb	1 (1.0)	1 (1.0)	0	

Perioperative complications classified by surgical approach

We summarized the perioperative complications according to the surgical approach because it can potentially influence the frequency of perioperative complications (Tables 2-4). In cases undergoing median sternotomy, complications, such as phrenic nerve palsy and arrhythmia, were observed in the non-GS group. In contrast, no perioperative complications were noted in the two GS cases (Table 2). In the cases treated with VATS, one GS patient developed a perioperative infection, which was successfully managed with antibiotic therapy (Table 3). Among the three GS cases treated with RATS, no early postoperative complications were observed (Table 4). No definitive conclusions could be drawn regarding differences in complication rates according to surgical approach due to the limited number of GS cases. Table 5 summarizes the univariate logistic regression analysis of the association between tumor diameter and perioperative complications across all surgical procedures. Regarding perioperative infection, no statistically significant association with tumor diameter was observed (p=0.185). However, it is noteworthy that all five cases of infection occurred in patients with tumors exceeding 5 cm in diameter.

**Table 2:** Postoperative complications of the cases who underwent surgery with median sternotomy Continuous variables are presented as medians and ranges. Counts are given as numbers. * Values in parentheses indicate the number of Clavien-Dindo grade III or higher complications. Abbreviations: GS, Good's syndrome; NA, Not applicable

Characteristics		Overall (n=33)	Non-GS (n=31)	GS (n=2)	p-value
Surgical procedure, n	Extended thymectomy	18	17	1	0.67
	Partial thymectomy	6	6	0	
	Total thymectomy	9	8	1	
Operation time (minutes), median (range)		225 (98-732)	226 (98-732)	195 (194-196)	0.65
Blood loss (mL), median (range)		200 (<5-2980)	200 (<5-2980)	200 (130-270)	0.91
Drainage duration (days), median (range)		3 (1-9)	3 (1-9)	1 (1-1)	0.052
Arrhythmia, n		4	4 (1) *	0	1
Infectious disease, n		3	3	0	1
Phrenic nerve palsy, n		6	6 (2) *	0	1
Recurrent laryngeal nerve palsy, n		1	1	0	1
Pleural effusion, n		3	3 (3) *	0	1
Atelectasis, n		4	4	0	1
Chylothorax, n		1	1	0	1

**Table 3:** Postoperative complications of the cases who underwent video-assisted thoracoscopic surgery Continuous variables are presented as medians and ranges. Counts are given as numbers. * Values in parentheses indicate the number of Clavien-Dindo grade III or higher complications. Abbreviations: GS, Good's syndrome; NA, Not applicable

Characteristics		Overall (n=37)	Non-GS (n=35)	GS (n=2)	p-value
Surgical procedure, n	Extended thymectomy	12	11	1	0.11
	Partial thymectomy	21	21	0	
	Total thymectomy	4	3	1	
Operation time (minutes), median (range)		130 (53-382)	118 (53-382)	206.5 (206-207)	0.24
Blood loss (mL), median (range)		<5 (<5-100)	<5 (<5-100)	50 (50-50)	<0.01
Drainage duration (days), median (range)		1 (1-5)	1 (1-5)	1 (1-1)	0.29
Arrhythmia, n		0	0	0	NA
Infectious disease, n		2	1	1	0.21
Phrenic nerve palsy, n		3	3 (1) *	0	1
Recurrent laryngeal nerve palsy, n		1	1	0	1
Pleural effusion, n		1	1	0	1
Atelectasis, n		0	0	0	NA
Chylothorax, n		0	0	0	NA

**Table 4:** Postoperative complications of the cases who underwent robotic-assisted thoracoscopic surgery Continuous variables are presented as medians and ranges. Counts are given as numbers. * Values in parentheses indicate the number of Clavien-Dindo grade III or higher complications. Abbreviations: GS, Good's syndrome; NA, Not applicable

Characteristics		Overall (n=34)	Non-GS (n=31)	GS (n=3)	p-value
Surgical procedure, n	Extended thymectomy	1	0	1	<0.01
	Partial thymectomy	23	22	1	
	Total thymectomy	10	9	1	
Operation time (minutes), median (range)		159.0 (57-335)	160.0 (57-335)	152 (90-283)	0.98
Blood loss (mL), median (range)		<5 (<5-250)	<5 (<5-250)	<5	0.37
Drainage duration (days), median (range)		2 (1-5)	2 (1-2)	2 (1-2)	0.59
Arrhythmia, n		0	0	0	NA
Infectious disease, n		0	0	0	NA
Phrenic nerve palsy, n		4	4	0	1
Recurrent laryngeal nerve palsy, n		0	0	0	NA
Pleural effusion, n		1	1 (1) *	0	1
Atelectasis, n		0	0	0	NA
Chylothorax, n		0	0	0	NA

**Table 5:** Univariate logistic regression analyses of the association between tumor diameter and perioperative complications Odds ratio were calculated for each 1-mm increase in tumor diameter. P-values were derived from univariate logistic regression models.

Complication	Odds Ratio	95% Confidence Interval	p-value
Arrythmia	1.025	0.988-1.069	0.194
Infectious disease	1.023	0.990-1.061	0.185
Phrenic nerve palsy	1.020	0.997-1.044	0.094
Recurrent laryngeal nerve palsy	1.010	0.956-1.067	0.693
Pleural effusion	1.022	0.989-1.060	0.201
Atelectasis	1.045	1.004-1.100	0.049
Chylothorax	1.077	0.988-1.256	0.189

Comorbidities and prognosis of the cases with GS

Table 6 summarizes the comorbid conditions and prognosis observed in seven GS cases. Of these, six patients had a history of recurrent infections before surgery. Regarding autoimmune diseases, two cases were complicated by MG, and three by postoperative pure red cell aplasia (PRCA). The time from surgery to PRCA onset was 5, 12, and 20 months for Patients 2, 4, and 5, respectively. Prophylactic sulfamethoxazole-trimethoprim had been initiated preoperatively and was maintained throughout the perioperative period in Patients 4 and 5. For the remaining patients, no postoperative prophylactic antibiotics were administered, following our standard protocol for thymic tumor resection. Six out of seven cases received preoperative intravenous immunoglobulin, and severe perioperative infectious diseases were not shown. No cases demonstrated tumor invasion into the mediastinal pleura based on histopathological assessment. Two patients died of extrathymic cancer that developed more than four years after surgery, and two patients died from severe cytomegalovirus infection. Figure 1 displays Kaplan-Meier survival curves comparing GS and non-GS groups. Although there is a marked disparity in sample size between the cohorts, the GS group demonstrated significantly poorer prognosis (p=0.002).

**Table 6:** Characteristics and prognosis of the patients with Good's syndrome Abbreviations: MS, Median sternotomy; VATS, Video-assisted thoracoscopic surgery; RATS, Robotic-assisted thoracoscopic surgery; CMV, Cytomegalovirus; SLE, Systemic lupus erythematosus; PRCA, Pure red cell aplasia; MG, Myasthenia gravis; NA, Not applicable; IVIG, Intravenous immunoglobulin; y, years; m, months

Patient number	1	2	3	4	5	6	7
Surgical approach	MS	VATS	MS	VATS	RATS	RATS	RATS
Age at operation (years old)	53	66	57	68	62	58	73
Sex	Male	Female	Male	Male	Male	Male	Male
Preoperative infectious disease	Pneumonia	Bronchiolitis	None	*Pneumocystis *pneumonia	*Pneumocystis *pneumonia	Pharynx candidiasis	Mandibular osteomyelitis
	*Candida *esophagitis			CMV pneumonia	CMV gastritis		
				*Candida *stomatitis	CMV stomatitis		
				*Candida *endophthalmitis	CMV uveitis		
Autoimmune syndrome	None	SLE	None	PRCA (postoperative)	PRCA (postoperative)	MG (asymptomatic)	None
		MG (asymptomatic)					
		PRCA (postoperative)					
Laboratory data at diagnosis/after surgery							
IgA (mg/dL) (Normal range: 93-393)	30/18	304/NA	2/6	198/48	47/33	7/<12	45/25
IgG (mg/dL) (Normal range: 861-1747)	319/744	562/NA	319/299	652/726	175/1391	400/701	541/678
IgM (mg/dL) (Normal range: 33-183)	<30/0	11/NA	25/73	10/19	10/9	4/<3	8/8
Lymphocyte subsets (%) CD4/CD8/CD20/CD4:CD8 ratio	29.4/35.4/0/0.83	NA	36.9/30.5/13.1/1.21	45.6/27.4/0.3/1.66	44.7/44.6/NA/1.00	31.1/59.9/NA/0.52	32.2/22.6/NA/1.42
Perioperative IVIG	15 g (261 mg/kg)	Not done	20 g (340 mg/kg)	15 g (272 mg/kg)	12 g (164 mg/kg)	20 g (292 mg/kg)	20 g (328 mg/kg)
Operation time (min)	194	207	196	206	152	283	90
Blood loss (mL)	270	50	130	50	<10	<10	<10
Histological type	AB	AB	AB	AB	AB	AB	AB
Tumor size (cm)	10.0 × 9.0 × 5.0	6.5 × 6.5 × 3.5	9.0 × 6.0 × 4.5	7.0 × 6.0 × 4.0	6.2 × 4.0 × 3.5	9.5 × 8.0 × 6.0	5.5 × 5.0 × 3.5
TNM/Masaoka-Koga classification	pT1aN0M0/IIb	pT1aN0M0/IIa	pT1aN0M0/I	pT1aN0M0/IIb	pT1aN0M0/IIa	pT1aN0M0/I	pT1aN0M0/IIa
Postoperative hospital stay (days)	6	5	7	5	5	8	8
Late postoperative infectious disease	CMV colitis	CMV colitis	*Candida *esophagitis	Chronic sinusitis	*Pneumocystis *pneumonia	None	None
	CMV retinitis	CMV retinitis	*Candida *stomatitis		CMV enteritis		
Postoperative follow-up period	14 y 9 m	8 y 5 m	6 y 4 m	4 y 6 m	5 y 8 m	2 y 7 m	1 y 10 m
Outcome	Died of CMV infection	Died of CMV infection	Died of maxillary sinus cancer	Died of lung cancer	Survival	Survival	Survival

## Discussion

Thymomas are well-known mediastinal diseases that are sometimes complicated by several autoimmune manifestations, such as MG, PRCA, and hypogammaglobulinemia. Previous studies reported that hypogammaglobulinemia occurs in 6-11% of patients with thymoma [1]. Over 90% of the patients experience repeated infections, such as pneumonia with B-cell depletion and inversion of the CD4/CD8 ratio [2,3]. One past study reported that the 10-year survival rate for GS was 33% [11]. Although recent studies reported that it was 53.7-84%, the prognosis is still poorer than that of other common immunodeficiency diseases [2,4]. Despite the rapid advancements in treatments for thymoma, there remain numerous uncertainties regarding the appropriate management and treatment of GS.

In this report, all seven cases of GS were diagnosed with type AB thymoma. Previous studies have reported that 41.7-59% of GS patients had type AB thymoma [1,3,4]. There were no differences in the pathological stage or tumor size between non-GS and GS cases. Although there was a significant bias in the number of cases, there was no tendency for longer operation time, and no significant perioperative complications were observed in GS patients. The risk of surgical intervention itself was considered to be comparable to that of thymoma without GS, and with the tailored use of antibiotics and intravenous immunoglobulin based on preoperative immunoglobulin levels and the presence or suspicion of infection, perioperative management was deemed safe.

However, it is reported that hypogammaglobulinemia rarely improves postoperatively [1,3], and in the present study, no cases showed refinement in immunodeficiency. Although the observation period was short, one case developed a severe infection postoperatively in the RATS group. While perioperative management could be safely achieved, the long-term risk of severe infections may have remained. Furthermore, two cases of GS developed extrathymic malignancies in the late postoperative period and subsequently died due to those cancers. There could be an increased risk of developing extrathymic malignancies after thymectomy, which might be related to T-cell dysfunction [12,13]. On the other hand, the patients with GS show marked immunodeficiency even before surgery. Although the influence of thymectomy on the risk of developing secondary malignancies in GS patients remains unclear, careful long-term monitoring for potential susceptibility to malignant diseases might be warranted. Possibly due to the elevated risk of severe infection and other malignancies in GS patients, survival analysis showed significantly poorer postoperative prognosis, suggesting no marked difference in long-term outcomes based on the surgical approach.

In this report, three cases developed PRCA postoperatively. Approximately 2-5% of thymoma cases are reported to be associated with PRCA [14], but co-occurrence of hypogammaglobulinemia and PRCA is even rarer, with only 14 documented cases over the past 20 years detailing their clinical course [15]. Among these, only two cases developed PRCA after detection of the thymoma [16,17]. Moreover, the development of PRCA occurring long after surgery is exceedingly rare [18]. Nevertheless, long-term postoperative surveillance is essential to monitor not only for infections but also for the development of other autoimmune diseases, including PRCA, and extrathymic malignancies, with regular assessment of serum immunoglobulin levels and periodic whole‑body imaging to detect these complications at an early stage.

This study has several limitations. First, the comparison between GS and non‑GS patients is inherently imbalanced, and the small number of GS cases limits the stability of statistical analyses and precludes meaningful adjustment for confounders. Nevertheless, all confirmed causes of death were infections or secondary malignancies, which remain closely linked to the underlying immunodeficiency of GS. Second, although the feasibility of VATS and RATS was demonstrated, conclusions regarding their comparative safety are preliminary because only a few GS patients underwent minimally invasive surgery. Moreover, the choice of surgical approach was influenced by the operating surgeon's experience, proficiency, and individual preference, and therefore selection bias cannot be fully excluded. Nevertheless, the procedures performed represented the standard surgical options for thymoma in Japan at the time each operation was performed. Third, perioperative management was not fully standardized, and the long study period may have introduced heterogeneity in surgical techniques and follow‑up practices. Although minimally invasive approaches and perioperative care have advanced over the past two decades, it remains unclear whether these developments have translated into improved long‑term outcomes in GS, and careful lifelong surveillance is still required for this challenging condition.

## Conclusions

Thymectomy for GS can be performed with acceptable perioperative safety, including minimally invasive approaches. However, regardless of the surgical procedures, there remains a risk of severe infections and the development of new malignancies late postoperatively, contributing to poorer long-term prognosis.
